# A comparison of the clinicopathological features and prognoses of the classical and the tall cell variant of papillary thyroid cancer: a meta-analysis

**DOI:** 10.18632/oncotarget.14055

**Published:** 2016-12-21

**Authors:** Zeming Liu, Wen Zeng, Tianwen Chen, Yawen Guo, Chao Zhang, Chunping Liu, Tao Huang

**Affiliations:** ^1^ Department of Breast and Thyroid Surgery, Union Hospital, Tongji Medical College, Huazhong University of Science and Technology, Wuhan, China; ^2^ Department of Ophthalmology, Zhongnan Hospital, Wuhan University, Wuhan, Hubei, China; ^3^ Department of Cardiovascular Surgery, Union Hospital, Tongji Medical College, Huazhong University of Science and Technology, Wuhan, China

**Keywords:** papillary thyroid carcinoma, tall cell variant, clinicopathological features, mortality risk, meta–, analysis

## Abstract

Papillary thyroid cancer (PTC) accounts for 80–90% of all thyroid malignancies. The tall cell variant (TCV) is a rare aggressive histotype of PTC. We performed a meta-analysis to compare the clinicopathological characteristics and prognostic factors of TCV with those of classical papillary thyroid carcinoma (cPTC). A literature search was performed using the PubMed and EMBASE databases using Medical Subject Headings and keywords. Twenty studies that included 1871 patients with TCV and 75323 patients with cPTC were included in our meta-analysis. Odds ratios and confidence intervals were calculated for each study. Patients with TCV were associated with multifocality, higher TNM stage, extrathyroidal extension, vascular invasion, lymph node metastasis, distant metastasis, *BRAF* mutation, disease-specific survival, and overall survival. We found that TCV cases were associated with more aggressive clinicopathological characteristics and poorer prognoses than cPTC cases were. Our results suggest that TCV is a high-risk PTC that warrants aggressive treatment and follow-up strategies.

## INTRODUCTION

Papillary thyroid cancer (PTC), whose global incidence has rapidly increased in recent decades, accounts for more than 80% of all thyroid carcinomas, making it the most common type of thyroid malignancy [1, 2]. PTC is derived from the follicular epithelium [3] and includes many histological variants such as tall cell, columnar cell, diffuse sclerosing, solid, and hobnail [4, 5].

The tall cell variant (TCV), a rare histological subtype of PTC that was first reported by Hawk et al. in 1976 [6], constitutes 5 to 11% of all PTC cases [7]. Kazaure et al. found that TCV incidence increased by 158% (0.05 per 100 000 to 0.13 per 100 000) between 2001 and 2008 [8]. Unlike classic PTC (cPTC), TCV tumors comprise obvious rectangular cells, with less colloid, but with nuclear features similar to those observed in cPTC [9]. TCV is usually defined as a PTC in which 30% or more of tumor cells are twice as long as they are wide; however, the World Health Organization defines PTC as TCV when the tumor is composed predominantly of cells whose heights are at least 3 times their widths [9].

Studies on the clinicopathological characteristics and prognostic outcome of TCV have had controversial results [10, 11]. No differences were found in some studies between the prognoses of TCV and cPTC, [10] whereas results of other studies and the current American Thyroid Association Guidelines for Differentiated Thyroid Cancer indicate that TCV displays more aggressive pathological characteristics at diagnosis and a poorer prognosis than cPTC does [1, 12]. Therefore, to resolve these discordant findings, we performed a meta-analysis to compare the clinicopathological characteristics at presentation, prognostic factors in terms of cancer-related death, and overall survival in TCV with that in cPTC.

## RESULTS

### Literature searches and study features

**Figure 1:**
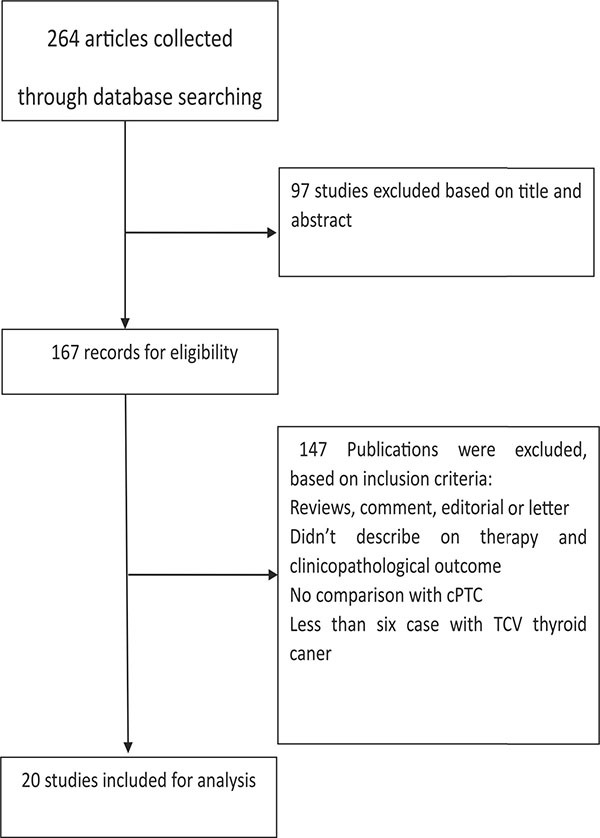
Flow chart showing the process of study selection for the meta–analysis

**Figure 2:**
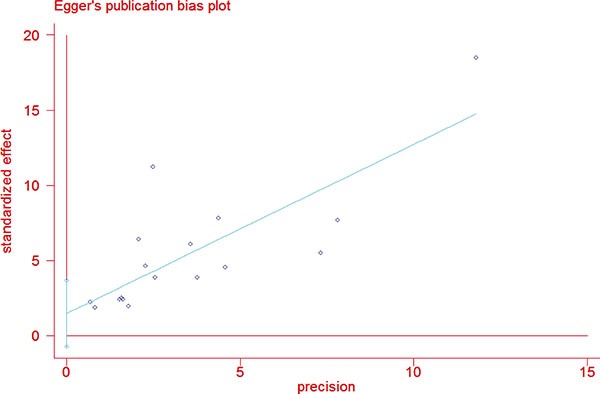
Funnel plots for publication bias considering both extrathyroidal extension

The study selection process is described in Figure 1. A total of 264 abstracts and titles were acquired by electronic searches. Of these primary selected abstracts and papers, 167 full-text articles were considered relevant and examined in detail. Following this detailed review, 20 studies that included a total of 1871 patients with TCV and 75323 patients with cPTC were selected using the described search strategy [8, 10–28]. The major features of the 20 selected studies, which included clinicopathological characteristics and prognostic factors, are summarized in Table 1. Of these 20 studies, 13 evaluated multifocality, 11 evaluated TNM stage, 17 reported on extrathyroidal extension (ETE), 7 evaluated vascular invasion, 19 assessed lymph node metastases (LNM), 10 reported on distant metastases (DM), 4 assessed *BRAF* mutation, 7 reported disease-specific survival, and 4 evaluated overall survival. The funnel plots for each outcome indicated no publication bias. Figure 2 shows the funnel plots for ETE (Figure 2A) and LNM (Figure 2B). Egger's linear regression analysis for ETE (*p* = 0.181) and LNM (*p* = 0.075) revealed no substantial asymmetry.

**Table 1:** Characteristics of studies included in the meta-analysis

Study	Year	Country	Ethnicity (A: Asians, C: Caucasians)	Study type	TCV number: cPTC number	Age (y) mean ± SD or median (range)	Female: Male	Follow-up, months	131I in TCV (%)	Poor Outcome
Hadiza	2012	USA	C	Retrospective	573:42904	55.3 (0.7); 47.1 (0.1)	413:160; 32779:10125	25.2; 64.8	56.8	Disease-specific survival, Overall survival
Eric	2013	USA	C	Retrospective	97:18260	49.6 (1.4) a; 47.6 (0.1)	78:17; 15009:3251	45.6; 63.3	39.2	Disease-specific survival, Overall survival
Oh	2014	Korea	A	Retrospective	95:203	47.1; 47.6	83:13; 164:39	NA	NA	NA
Nardone	2003	USA	C	Retrospective	17:12	54.1 ± 14.4; 34.3 ± 11.7	7:5; 10:7	NA	NA	NA
Alex	2008	China	A	Retrospective	14:1094	53.7 (33–81); 45.2 (7–94)	10:4; 891:203	NA	92.9	Recurrence, cause-specific survival
Okuyucu	2015	Turkey	C	Retrospective	70:862	49.1; 39.9	46:24; 652:210	146.2 ± 43.7	100	Recurrence
Lee	2013	Korea	A	Retrospective	13:202	54.2; 44.8	13:0;160:42	NA	NA	NA
Ito	2008	Korea	A	Retrospective	60:1313	NA	57:3; 1218:97	154.8;154.8	NA	Disease-free survival, cause-specific survival
Michels	2006	France	C	Retrospective	56:503	50; 45.6	47:9; 416:87	84; 84	67	Disease-specific survival
Ganly	2014	USA	C	Retrospective	134:288	NA	89:45; 211:77	112;112	74	Disease-specific survival, recurrence-free survival
Prendiville	2000	USA	C	Retrospective	20:1355	49.6; 35.7	NA	45.6;189.6	NA	Cancer-related mortality
Bernstein	2013	USA	C	Retrospective	27:26	56; NA	24:2; 22:5	20;20	69.2	Disease-free survival
Morris	2010	USA	C	Retrospective	278:2522	54.3; 46.3	207:71; 1864:658	28.0;26.1	55.0	Disease-specific survival
Axelsson	2014	Iceland	C	Retrospective	49:327	66; 49	29:20; 258:69	92.4; 130.8	NA	Disease-specific survival, Overall survival
Ghossein	2007	USA	C	Retrospective	62:83	41; 39	51:11;65:18	33.6;36	63.3	Recurrence
Beninato	2013	USA	C	Retrospective	59:58	45.1 ± 13.7; 44.9 ± 13.9	44:15; 47:11	30;20	93	Recurrence
Machens	2004	Germany	C	Retrospective	16:316	57; 46	10:6; 231:85	NA	NA	NA
Regalbuto	2013	NA	C	Retrospective	30:293	50.6 ± 12.8; 47.3 ± 13.2	25:5; 250:43	89;89	NA	Persistent or recurrent disease
Min	2013	Korea	A	Retrospective	23:303	47.8; 55.1	20:3;249:54	33.1;33.1	NA	NA
Shi	2016	USA	C	Retrospective	239:4702	51 (39–64) 43 (33–55)	174:65 3584:1118	37.0	89.1	Recurrence, Overall survival

^a^Standard error of the mean (SEM); NA: not available.

### Meta-analysis of clinicopathological features at diagnosis: TCV vs. cPTC

**Figure 3:**
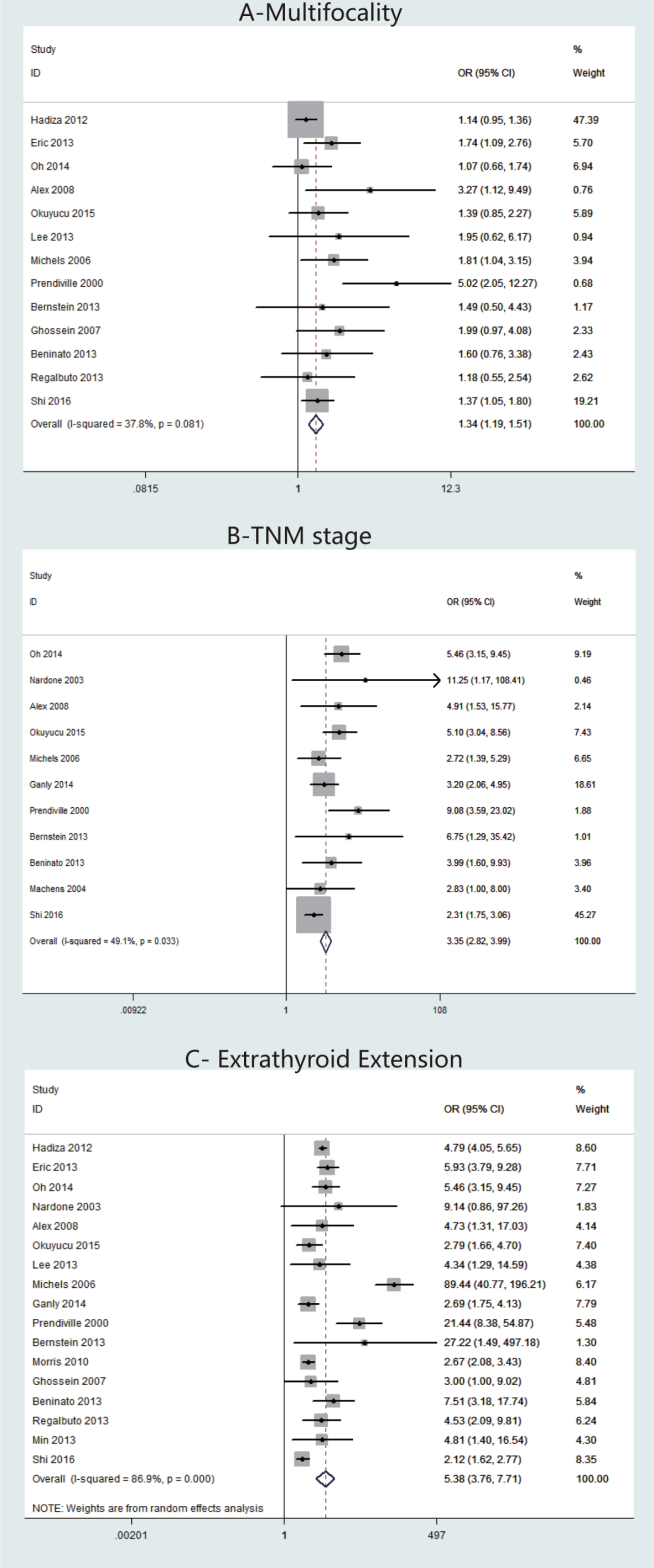
Forest plots of odds ratios (ORs) for multifocality (Panel **A**), TNM stage (Panel **B**) and extrathyroidal extension (Panel **C**) associated with the tall cell variant (TCV) vs classic papillary thyroid cancer (cPTC). Each study is represented as a square and a horizontal line: the area of the square reflects the weight of the study in the meta-analysis, while the line represents the OR with its confidence interval. Diamonds represent the pooled ORs and their confidence interval.

Thirteen studies presented clinical data on multifocality (Figure 3A). A fixed-effects model was adopted because heterogeneity was not significant between multifocality and TCV (*P* = 0.081, *I*^2^ = 37.8%). The OR from the 13 studies was 1.34 (95% CI = 1.19–1.51). Our analysis revealed that the occurrence of multifocality in TCV was significantly higher than that in cPTC (*P* < 0.001).

TNM stage was reported for patients in 11 studies (Figure 3B). A fixed-effects model was adopted because heterogeneity was not significant between TNM stage and TCV (*P* = 0.033, *I*^2^ = 49.1%). The OR from the 11 studies was 3.35 (95% CI = 2.82–3.99). Our analysis revealed that the TNM stage in TCV was significantly higher than that in cPTC (*P* < 0.001).

Regarding ETE cases, 17 studies were included (Figure 3C). A random-effects model was adopted because heterogeneity was significant between ETE and histology types (*P* < 0.00001, *I*^2^ = 86.9%). According to our analysis, ETE occurred more frequently in patients with TCV than in patients with cPTC. The overall OR was 5.38 (95% CI = 3.76–7.71, *P* < 0.001). We assessed the studies individually by sequentially excluding each of the 17 studies from our meta-analysis. Using this method, we found that *I*^2^ decreased to 75.4% when we excluded the study by Michels et al. [18]; therefore, we concluded that the heterogeneity was mainly caused by this particular study (data not shown).

**Figure 4:**
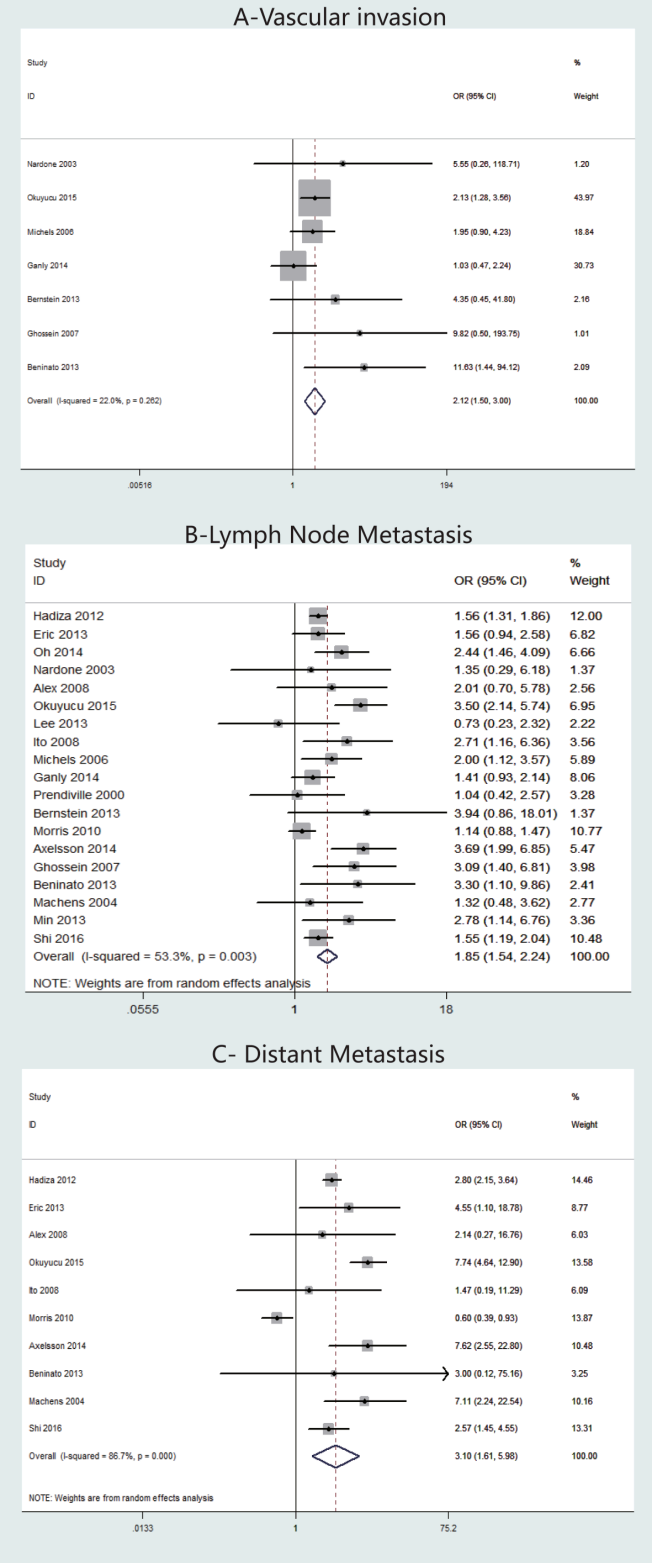
Forest plots of odds ratios (ORs) for vascular invasion (Panel **A**), lymph node metastasis (Panel **B**) and distant metastasis (Panel **C**) associated with the tall cell variant (TCV) vs classic papillary thyroid cancer (cPTC). Each study is represented as a square and a horizontal line: the area of the square reflects the weight of the study in the meta-analysis, while the line represents the OR with its confidence interval. Diamonds represent the pooled ORs and their confidence interval.

Seven studies presented clinical data on vascular invasion (Figure 4A). A fixed-effects model was adopted, because heterogeneity was not significant between vascular invasion and TCV (*P* = 0.262, *I*^2^ = 22%). The OR from the 12 studies was 2.12 (95% CI = 1.50–3.00). Our analysis revealed that the occurrence of vascular invasion in TCV was significantly higher than that in cPTC (*P* < 0.001).

LNM stage was reported for patients in 19 studies (Figure 4B). A random-effects model was adopted because heterogeneity was significant between TNM stage and TCV (*P* = 0.003, *I*^2^ = 53.3%). The OR from the 19 studies was 1.85 (95% CI = 1.54–2.24). Our analysis revealed that the occurrence of LNM in TCV was significantly higher than that in cPTC (*P* < 0.001). We assessed the studies individually by sequentially excluding each of the 19 studies from our meta-analysis. Using this method, we found that *I*^2^ decreased to 41.8% when we excluded the study by Okuyucu et.al [11], therefore, we concluded that the heterogeneity was mainly caused by this particular study (data not shown).

Regarding DM cases, 10 studies were included (Figure 4C). A random-effects model was adopted because heterogeneity was significant between DM and histology types (*P* < 0.00001, *I*^2^ = 86.7%). According to our analysis, DM occurred significantly more frequently in patients with TCV than in patients with cPTC. The overall OR was 3.10 (95% CI = 1.61–5.98).

**Figure 5:**
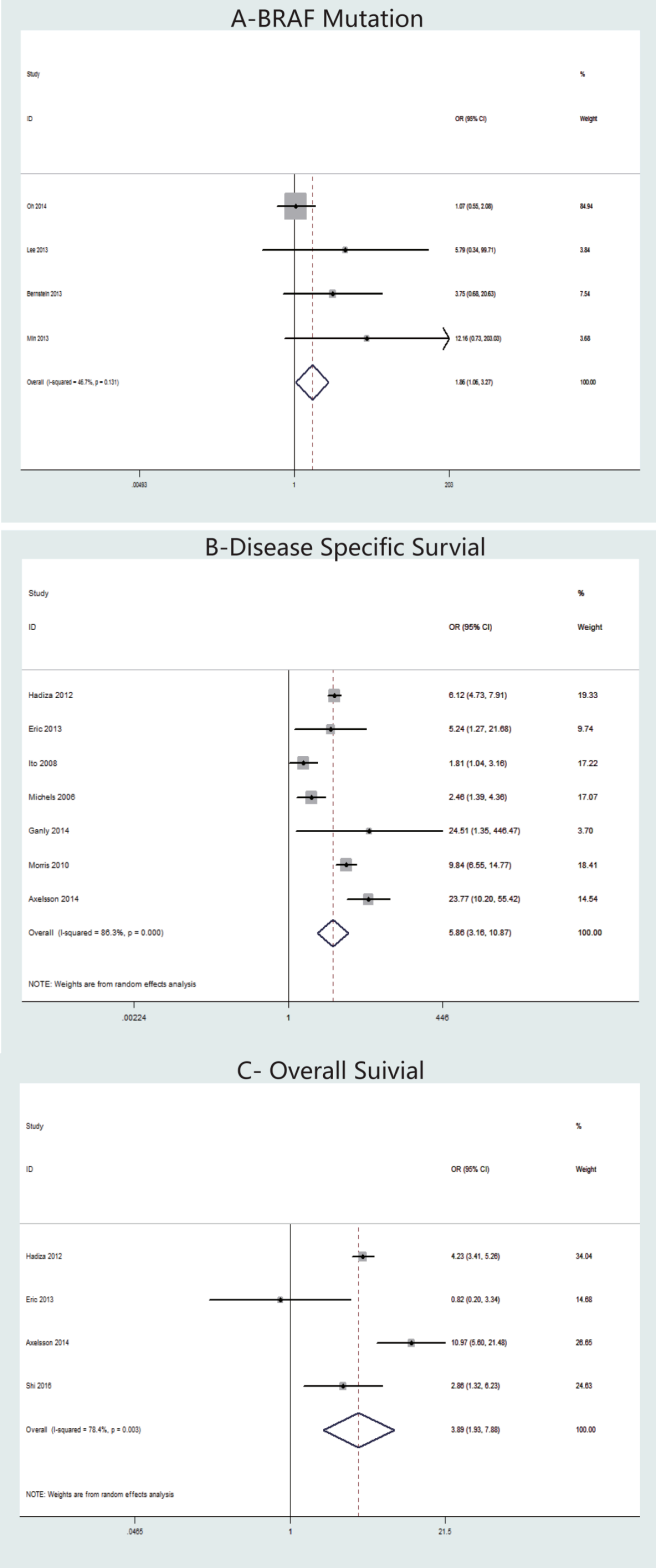
Forest plots of odds ratios (ORs) for *BRAF* mutation (Panel **A**), disease-specific survival (Panel **B**) and overall survival (Panel **C**) associated with the tall cell variant (TCV) vs classic papillary thyroid cancer (cPTC). Each study is represented as a square and a horizontal line: the area of the square reflects the weight of the study in the meta-analysis, while the line represents the OR with its confidence interval. Diamonds represent the pooled ORs and their confidence interval.

Concerning the presence of *BRAF* mutation, four studies were included in the meta-analysis (Figure 5A). A fixed-effects model was adopted because heterogeneity was not significant between *BRAF* mutation and TCV (*P* = 0.131, *I*^2^ = 46.6%). The OR from the 12 studies was 1.86 (95% CI = 1.06-3.27). According to our analysis, the occurrence of *BRAF* mutation in TCV was significantly higher than that in cPTC (*P* = 0.030).

### Meta-analysis of disease-specific survival and overall survival: TCV vs. cPTC

Seven studies were included in our meta-analysis of disease-specific survival (Figure 5B). We adopted a random-effects model because the heterogeneity of the data was significant (*P* < 0.00001), and the *I*^2^ estimate of the variance between the studies was 86.3%. Our analysis showed that disease-specific death was significantly more frequent in patients with TCV than in those with cPTC (OR = 5.86, 95% CI = 3.16–10.87; *p* < 0.001).

Mortality data were reported in four studies (Figure 5C). A random-effects model was adopted, because the heterogeneity between mortality and histology types was significant (*P* = 0.003, *I*^2^ = 78.4%). Our analysis revealed that the overall mortality in TCV was significantly higher than that in cPTC; the overall OR was 3.89 (95% CI = 1.93–7.88, *P* < 0.001).

### Subgroup analyses of the effects of TCV on aggressive clinicopathological features and prognostic factors

We performed subgroup analysis according to the patients’ ethnicities in order to investigate ethnicity as a potential source for heterogeneity, and to determine whether the effects of TCV on aggressive clinicopathological features and poor prognosis of PTC were associated with patient ethnicity (Table 2). The effect estimates were broadly consistent among the analyzed subgroups. Heterogeneity was markedly decreased in the subgroup analyses of LNM, but not in the subgroup analyses of ETE, DM, and disease-specific survival.

**Table 2:** Subgroup analysis according to patient ethnicity of the effects of the tall cell variant on the aggressive clinicopathological features and poor prognosis of papillary thyroid cancer

Subgroup	Odds ratio	95% confidence interval	*I^2^* (%)	Model used
Extrathyroidal extension				
Asians (3 studies)	5.17	[3.24, 8.25]	0	Fixed-effects
Caucasians (14 studies)	5.51	[3.67, 8.28]	89.3	Random-effects
Lymph node metastases				
Asians (6 studies)	2.55	[1.91, 3.39]	21.6	Fixed-effects
Caucasians (13 studies)	1.64	[1.37, 1.98]	43.6	Random-effects
Distant metastasis				
Asians (2 studies)	1.74	[0.41, 7.41]	0	Fixed-effects
Caucasians (8 studies)	3.36	[1.64, 6.89]	89.6	Random-effects
Disease-specific survival				
Asians (1 study)	1.81	[1.04, 3.16]	-	-
Caucasians (6 studies)	7.34	[4.10, 13.13]	86.6	Random-effects

## DISCUSSION

TCV is usually diagnosed postoperatively on the basis of routine pathology because an accurate preoperative diagnosis is difficult. Classic TCV tumors are “composed of more than 50% tall cells, a tall cell height at least twice as long as its width, eosinophilic tall cell cytoplasm, and nuclear features characteristic of PTC such as nuclear irregularities, clearing and overlapping, grooves, and pseudoinclusions” [29]. Preoperative sonographic findings indicative of TCV often overlap significantly with those indicative of cPTC, but TCV often harbors features that are more aggressive, such as hypoechogenicity, evidence of ETE, microcalcifications, and macrocalcifications. Therefore, clinicians should consider a TCV diagnosis if such preoperative sonographic findings are present.

Michels et al. reported that TCV was associated with worse prognosis on univariate analysis but not on multivariate analysis [18]. They recommended that TCV should be considered a marker of more aggressive disease but that it was not an independent predictor of prognosis. However, according to Hadiza et al., patients with TCV had worse prognoses than patients with cPTC did, despite receiving relatively more radical treatment such as thyroid surgery and radiotherapy [8]. In our PTC series, patients with TCV displayed more aggressive tumor behavior, including multifocality, higher TNM stage, ETE, vascular invasion, LNM, and DM at diagnosis, and higher disease-specific mortality and overall mortality rates at follow-up than patients with cPTC did. Therefore, we recommend a more radical treatment strategy and close follow-up of patients with TCV.

The interest in molecular analysis of PTC has been growing for both diagnostic and prognostic reasons [30]. Molecular analysis typically involves a *BRAF* (V600E) mutation, which has emerged as a marker of aggressive behavior in thyroid cancer and is associated with clinical progression and recurrence of PTC [3]. The prevalence of *BRAF* mutation rates in TCV vary across reports. Several authors have reported a high prevalence of *BRAF* mutations in TCV, while others have reported a low prevalence [7, 10, 12, 16, 31]. In the present study, we demonstrated that *BRAF* mutations were associated with TCV. These discrepant findings may be because *BRAF* mutations occur in early stages of tumorigenesis and are associated with specific morphology and aggressive characteristics.

It is unknown whether aggressive tumor behavior is associated with the other molecular profiles of TCV. Some studies have indicated that molecular factors intrinsic to TCV are responsible for its aggressive biologic and clinical features [32]. For example, Wreesmann et al. and Campo et al. illustrated that the high prevalence of Muc1 and type IV collagenase expression may result in degradation of stroma and greater invasive properties in TCV compared to those in cPTC and its follicular variant [33, 34].

TCV cases that were treated with total thyroidectomy and accessorial radioactive iodine (RAI) therapy had better survival rates than cases with lower rates of surgical resection and no RAI therapy did [9]. However, Ghossein and Livolsi found that TCV is overrepresented in fluorodeoxyglucose positron-emission tomogram (FDG-PET)-positive thyroid carcinomas that are refractory to RAI therapy, which may due to a high prevalence of *BRAF* somatic mutations in patients with TCV [35]. Furthermore, Rivera et al. found that 20% of FDG-PET-positive/RAI therapy-refractory tumors are TCV tumors [36]. Therefore, more studies are needed to differentiate TCVs that are refractory to RAI therapy, and to develop effective targeted therapies against these otherwise incurable carcinomas.

A major limitation of this meta-analysis is the potential heterogeneity caused by differences in disease management practices, pathology reporting, follow-up duration, and the definition of remission, which may have affected the study conclusions. Another limitation is the small number of included articles and the unavailability of relevant unpublished data for further analysis. Therefore, larger studies would help address the role of TCV in the worsening of PTC prognosis in a more definite manner.

The present meta-analysis demonstrated that patients with TCV, an aggressive variant of PTC, present with more unfavorable features at diagnosis, such as multifocality, higher TNM stage, ETE, vascular invasion, LNM, DM, and *BRAF* mutations. Higher disease-specific mortality and overall mortality rates are found during follow-up in these patients than in patients with cPTC. The results of our meta-analysis demonstrate that TCV should be considered a high-risk PTC and that aggressive treatment and follow-up strategies should be adopted in these cases.

## MATERIALS AND METHODS

### Search strategy and literature selection

A systematical literature search was performed using online electronic databases (PubMed, EMBASE, and ISI Web of Science) for published papers through September 30, 2016, and the search was supplemented by manual searching and reference backtracking using the following Medical Subject Headings and keywords: “tall cell variant”, “TCV”, “thyroid,” “neoplasm(s),” “tumor,” “cancer,” and “carcinoma”. Relevant unpublished data that were presented at international meetings such as the American Thyroid Association meeting were also included. We contacted the authors for additional tabular data when necessary. The searches were limited to studies conducted in humans and written in English. Furthermore, the reference lists of retrieved articles were also reviewed to identify additional studies.

The following criteria were applied into the literature selection for studies that examined the association of TCV with high-risk clinic pathological factors and prognostic outcomes: (1) the studies had a randomized controlled trial or retrospective comparative study (cohort or case-control study) design; (2) the studies compared the TCV and cPTC groups of patients; (3) the study investigated at least one outcome of interest; and (4) weighted mean differences (WMDs) and ORs with 95% CIs were reported or were available to be calculated. Studies lacking a control population, duplicates of previous publication, animal studies, abstracts, single-case reports, and reviews were excluded. For studies with the same or overlapping data published by the same investigators, we selected studies with complete designs and larger sample sizes in our meta-analysis.

### Data extraction

All data were extracted independently by two authors (ZM Liu and W Zeng) and cross-checked to resolve any discrepancies. The following information regarding the association of TCV with clinicopathological risk factors, disease-related mortality, and overall mortality of PTC was extracted from each included study: first author, publication year, study location, ethnicity, number of TCV cases, age, female:male ratio in both TCV and cPTC groups, and the incidence rate of clinicopathological features, RAI therapy, and poorer outcome, in both TCV and cPTC groups.

The following outcomes were extracted for patients with primary PTC in order to compare the TCV and cPTC groups: the presence of ETE, larger tumor size, multifocality, LNM, DM, stage, vascular invasion, disease-specific survival, and overall mortality. All procedures conformed to the guidelines for the meta-analysis of observational studies in epidemiology [37].

### Statistical analysis

The summary ORs with 95% CIs and weighted mean differences with 95% CIs were calculated to compare dichotomous and continuous variables, respectively. The χ^2^-based Cochran's Q statistic test and *I*^2^ statistics were used to evaluate heterogeneity between the studies. Heterogeneity was considered significant when *P* was < 0.1 for the Q statistic or for an *I*^2^ statistic > 50% [38]. A fixed-effects model (Mantel-Haenszel method) was used when no significant heterogeneity was detected; otherwise, a random-effects model (DerSimonian-Laird method) was applied. Subgroup analyses were also performed according to patient ethnicity. In addition, we performed sensitivity analysis to assess the influence of each study on the overall estimate. Moreover, the potential publication bias was assessed using Egger's test and funnel plot analysis. All analyses were conducted using Stata (version 13.1; StataCorp LP, College Station, TX, USA). The *P*-values were two-tailed with the level of significance set at 0.05.
