# Correction: A comparison of the clinicopathological features and prognoses of the classical and the tall cell variant of papillary thyroid cancer: a meta-analysis

**DOI:** 10.18632/oncotarget.24998

**Published:** 2018-03-23

**Authors:** Zeming Liu, Wen Zeng, Tianwen Chen, Yawen Guo, Chao Zhang, Chunping Liu, Tao Huang

**Affiliations:** ^1^ Department of Breast and Thyroid Surgery, Union Hospital, Tongji Medical College, Huazhong University of Science and Technology, Wuhan 430022, China; ^2^ Department of Ophthalmology, Zhongnan Hospital, Wuhan University, Wuhan, Hubei, China; ^3^ Department of Cardiovascular Surgery, Union Hospital, Tongji Medical College, Huazhong University of Science and Technology, Wuhan, China

**This article has been corrected:** The proper name of the institution is as follows:

^1^ Department of Breast and Thyroid Surgery, Union Hospital, Tongji Medical College, Huazhong University of Science and Technology, Wuhan 430022, China

Original article: Oncotarget. 2017; 8:6222-6232. https://doi.org/10.18632/oncotarget.14055

